# Feasibility and Acceptability of Videoconference-Based Family Psychoeducation for Young Adult Caregivers of Family Members With Major Depressive Disorder: Protocol for a Pilot Randomized Controlled Trial

**DOI:** 10.2196/105182

**Published:** 2026-10-23

**Authors:** Hideki Nakamura, Sho Okawa, Hiroki Tanoue, Yoshihiro Goto, Akari Matsuzawa, Keiko Ito

**Affiliations:** 1 Department of Mental Health Nursing, Faculty of Nursing, Toho University, 4-16-20, Omori-Nishi, Ota-ku, Tokyo, 143-0015, Japan, 81 3-3762-9881, 81 3-3766-3914; 2 Department of Life Sciences, The University of Tokyo, Tokyo, Japan; 3 School of Nursing, Faculty of Medicine, University of Miyazaki, Miyazaki, Japan

**Keywords:** major depressive disorder, young adult caregivers, family caregivers, psychoeducation, videoconference-based intervention, telehealth, pilot randomized controlled trial, protocol, feasibility, acceptability

## Abstract

**Background:**

Young adult caregivers of a family member with major depressive disorder (MDD) constitute a vulnerable population. They have substantial support needs because they often experience psychological distress and daily burdens during a developmental period in which education, partnership formation, and parenting frequently overlap with caregiving responsibilities. Consequently, the burden associated with caring for a family member with MDD may adversely affect young adult caregivers’ mental health and life stability. Despite these needs, support programs and intervention studies specifically targeting young adult caregivers remain scarce. Videoconference-based family psychoeducation (vFPE) may help address this gap, improving access for individuals facing time and geographic barriers. However, the feasibility of conducting a randomized controlled trial (RCT) of vFPE in this population has not yet been established.

**Objective:**

The objective of this study is to examine the feasibility of conducting a waitlist-controlled pilot RCT of vFPE and the acceptability of the vFPE intervention among young adult caregivers of a family member with MDD.

**Methods:**

This study is a waitlist-controlled pilot RCT. Young adult caregivers (aged 18‐39 y) will be randomly assigned in a 1:1 ratio to either the intervention group or a waitlist control group. Participants in the intervention group will receive 4 weekly 40-minute vFPE sessions. The primary outcomes are feasibility and acceptability. Feasibility will be assessed by recruitment, randomization, intervention completion, dropout rates, and safety. Acceptability will be assessed using the Client Satisfaction Questionnaire-8 Japanese version. Exploratory outcomes, including anxiety and depressive symptoms, mental well-being, psychological distress, caregiver burden, self-efficacy, and health-related quality of life, will be assessed at baseline, postintervention, and 1-month follow-up via online self-administered questionnaires. Exploratory outcomes will be used to inform the design of a future definitive RCT rather than to draw conclusions about intervention effectiveness.

**Results:**

This paper describes the study protocol. Ethical approval was obtained from the Research Ethics Committee, Faculty of Nursing, Toho University, on December 18, 2025. Recruitment commenced on January 10, 2026, with the first participant enrolled on February 20, 2026. As of May 2026, 8 participants had been enrolled. Intervention delivery and data collection are expected to continue until March 2028. Study findings will be disseminated through presentations at academic conferences and publication in peer-reviewed journals.

**Conclusions:**

This study will clarify the feasibility of conducting an RCT of vFPE among young adult caregivers of a family member with MDD, providing foundational data to inform the design of a future definitive RCT.

## Introduction

### Background

Major depressive disorder (MDD) is a mental disorder that can have a persistent impact on both affected individuals and their family members [1]. Specifically, family caregivers often experience psychological distress and daily burdens while providing emotional support, assisting with daily activities, and managing crises [2,3].

Among these caregivers, young adults represent a particularly overlooked population [4,5]. Young adulthood is a developmental period characterized by major life transitions, such as achieving vocational independence, forming partnerships, and beginning parenthood. Therefore, assuming caregiving responsibilities for a family member with MDD during this stage may impose unique burdens distinct from those experienced in other life stages [6,7]. Furthermore, these responsibilities often conflict with developmental tasks and everyday obligations, potentially hindering access to support and help-seeking services. Accordingly, young adult caregivers of a family member with MDD are considered to have distinct support needs [8]. A recent meta-analysis of randomized controlled trials (RCTs) found that young people with a parent affected by serious physical or mental illness are at increased risk of psychological and developmental difficulties, and that psychosocial interventions have small but significant benefits for psychological outcomes and positive adjustment. However, young adults remain underrepresented in this evidence base, with only one trial specifically targeting young adults [9].

Family psychoeducation (FPE) has been recognized as a promising intervention for caregivers of a family member with MDD. Previous studies have suggested that FPE may reduce psychological burden and distress among family caregivers [10,11]. However, existing research has primarily focused on middle-aged and older spouses or parents, and intervention studies specifically targeting young adult caregivers remain limited [11,12]. Consequently, it remains unclear whether existing evidence regarding FPE can be generalized to young adult caregivers.

Furthermore, supporting young adult caregivers requires both intervention content and delivery modes that are compatible with work, study, parenting, and other responsibilities. In this regard, online support is a particularly suitable approach for young adult caregivers balancing multiple roles because it mitigates time-related and geographic barriers [13,14]. However, one-way online support focused solely on self-help or information provision may not sufficiently address an individual’s life circumstances and psychological needs. Accordingly, recent studies have highlighted the importance of interactive online support that incorporates responsive communication tailored to users’ situations and opportunities for interaction with support providers [15,16].

From this perspective, videoconference-based family psychoeducation (vFPE) is a promising approach for providing interactive online support to young adult caregivers. Videoconferencing allows real-time interaction with a supporter and may facilitate flexible responses tailored to individual family circumstances and life contexts [17,18]. Additionally, an individualized, rather than a group, format was selected to reduce potential concerns about confidentiality and discomfort associated with group participation [19].

We previously developed vFPE for young adult caregivers and evaluated it in an unpublished preliminary single-arm study involving 12 participants. The findings provided preliminary evidence of the feasibility, acceptability, and safety of vFPE. However, challenges specific to RCTs, such as randomization acceptance and retention at 1-month follow-up assessments, remain unexplored. Therefore, before proceeding to a definitive RCT, it is necessary to evaluate the feasibility of conducting a waitlist-controlled RCT of vFPE for young adult caregivers.

### Objective

This study aims to evaluate the feasibility of conducting a waitlist-controlled RCT of vFPE and the acceptability of the intervention among young adult caregivers of a family member with MDD. Specifically, it will assess recruitment, randomization, intervention completion, dropout rates, safety, and intervention acceptability to generate the foundational evidence to inform the design of a future definitive RCT.

## Methods

### Study Design

This study is a waitlist-controlled pilot RCT designed to examine the feasibility of conducting a trial of vFPE for young adult caregivers of a family member with MDD. The primary objective is to evaluate the feasibility of the trial procedures, including recruitment, randomization, intervention delivery, and a 1-month follow-up assessment, as well as the acceptability of the vFPE intervention. These evaluations will provide foundational data for a future definitive RCT. The secondary objective is to explore preliminary changes in clinical outcomes, particularly anxiety and depressive symptoms. This protocol follows the SPIRIT (Standard Protocol Items: Recommendations for Interventional Trials) 2025 guidelines (Checklist 1) [20].

### Eligibility Criteria

The eligibility criteria are as follows: (1) being aged 18 to 39 years, (2) having a spouse or a relative within the third degree of kinship who, according to participant-reported information, has been diagnosed with MDD by a physician, (3) providing care for that family member, and (4) being able to participate in the videoconference-based intervention using an internet connection and a computer or similar device.

In this study, the age range for young adult caregivers is broadly defined as 18 to 39 years. While the definitions of young adult caregivers vary across studies, this range was selected to encompass the Japanese life stages in which developmental tasks such as employment, career development, partnership, and parenting often overlap with family caregiving, reflecting the prolonged transition to adulthood [21]. This definition is also consistent with Japanese youth-support policies, in which people younger than 40 years may be included depending on the specific policy [22]. In addition, recent local government surveys of young carers in Japan have used 18 to 39 years as the target age range and reported that 4.8% to 6.8% of respondents were currently providing care for a family member [23,24]. Therefore, this study adopts the age range of 18 to 39 years to capture young adult caregivers with potential caregiving-related support needs. Participants will be spouses or relatives within the third degree of kinship to the family member with MDD, including siblings, children, and other relatives. The caregiver’s relationship to the family member with MDD will be recorded and summarized descriptively. In this study, “care” is not limited to physical assistance but broadly includes emotional support, assistance with daily living, and responses to psychological crises [25,26]. Therefore, eligibility will not be determined using rigid metrics for the specific types or duration of care; rather, the emphasis will be placed on whether individuals perceive themselves as having an ongoing supportive role.

The diagnosis and clinical background of the family member with MDD will be based on participant-reported information and will not be independently verified through direct clinical interviews, inquiries to physicians, or review of medical records to protect the family member’s privacy, reduce participant burden, and maintain the feasibility of recruitment in this pilot trial. To determine eligibility and reduce diagnostic uncertainty, the following information will be collected during eligibility screening based on information reported by the participant: (1) whether the family member has been diagnosed with MDD by a physician, (2) whether the participant has been directly informed of the diagnosis, (3) the time of diagnosis, (4) the duration of illness, (5) current psychiatric or mental health treatment status and frequency of visits, (6) history of past hospitalization, (7) major depressive symptoms currently experienced by the family member, and (8) any other known psychiatric diagnoses.

The exclusion criteria are as follows: (1) the participant is currently receiving outpatient treatment for a mental disorder, (2) the participant has a serious, progressive physical illness likely to hinder continued participation, or (3) the principal investigator determines that participation is difficult because of safety concerns, difficulty in understanding the study procedures, or substantial difficulty in maintaining participation. The first exclusion criterion was established because this pilot trial focuses on evaluating the feasibility of the trial procedures and the acceptability of vFPE under stable study conditions. Similar eligibility restrictions have been used in previous FPE trials for caregivers of people with mental illness [27]. Participants currently receiving outpatient treatment for a mental disorder may require individualized medical care or support beyond the scope of this study. Therefore, this criterion was intended to prioritize participant safety and reduce the potential influence of ongoing mental health treatment on the study findings.

### Recruitment

Participants will be recruited from January 2026 to December 2027 through administrative agencies, family associations, and online platforms. Recruitment will continue until the target sample size of 24 participants is reached or until December 2027, whichever occurs first. The number of participants at each stage of recruitment, reasons for ineligibility or nonparticipation, and recruitment sources will be recorded and summarized descriptively.

### Procedure

Interested individuals will be asked to contact the principal investigator directly using the method outlined in the study information materials. Those who contact the investigator will receive a comprehensive explanation of the study, covering its purpose, procedures, and ethical considerations via videoconferencing. Subsequently, written informed consent will be obtained.

After providing written informed consent, potential participants will provide the background and clinical information required for eligibility screening via an online self-administered questionnaire. The principal investigator will assess eligibility based on the predefined inclusion and exclusion criteria. Individuals determined to be eligible will then complete the baseline outcome questionnaires online and be enrolled in the study. The planned participant flow through the trial is illustrated in Figure 1.

**Figure 1.**
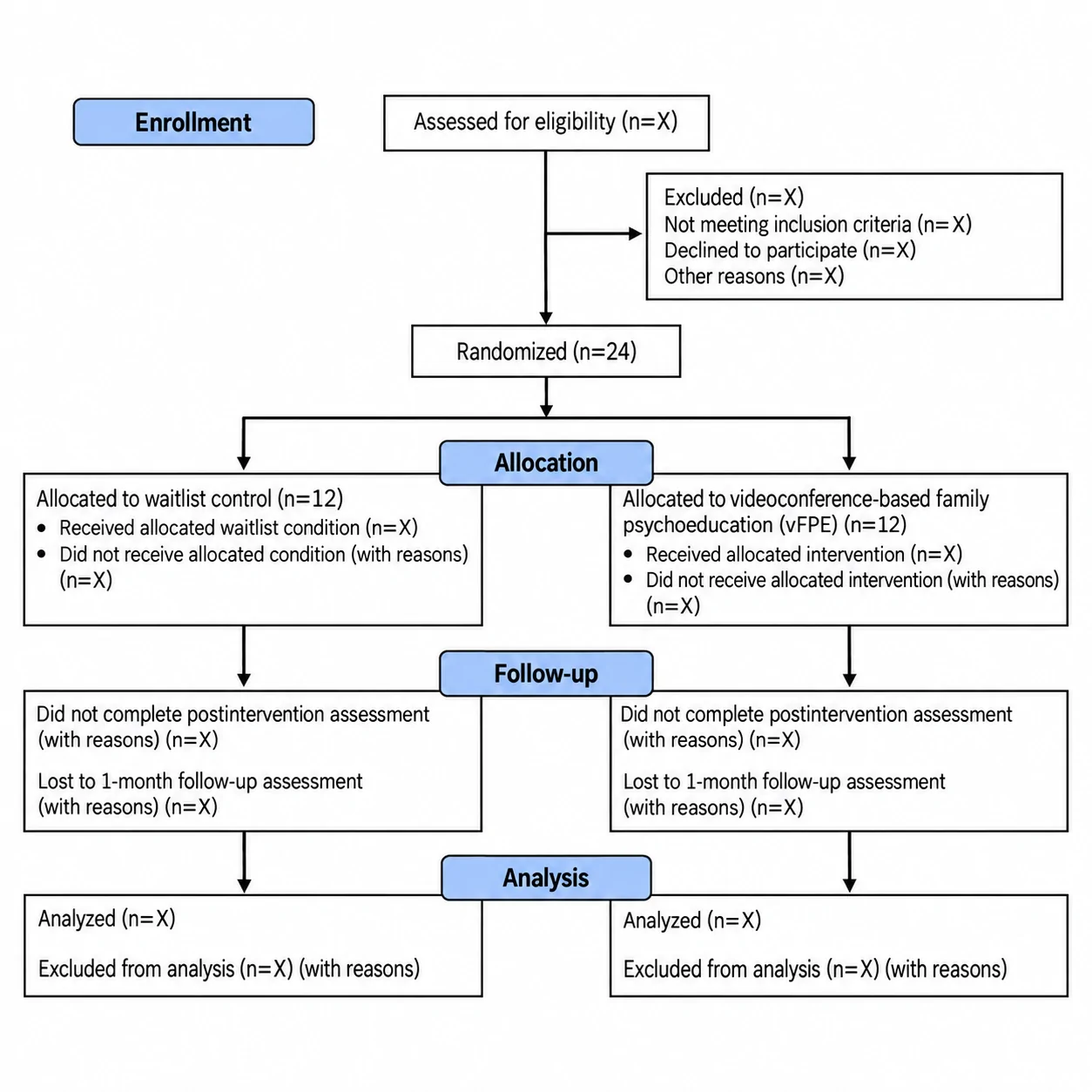
CONSORT (Consolidated Standards of Reporting Trials) flow diagram of participant recruitment, randomization, and retention. The diagram shows the planned participant flow through recruitment, eligibility screening, randomization, intervention, postintervention assessment, and 1-month follow-up assessment. The numbers shown for randomization and allocation represent the planned sample sizes; X indicates values that are not yet available and will be reported in the trial results.

### Intervention

#### vFPE Program

Participants in the intervention group will receive the vFPE program. This intervention will be delivered as individualized FPE sessions in a one-to-one format involving the intervention provider and the participant, and the family member with MDD will not attend the sessions. The program was developed using a coproduction framework to ensure that it reflects the support needs of young adult caregivers [28,29]. From the initial development stages, 2 young adult caregivers participated as co-designers, providing input on the challenges commonly encountered by this population, as well as the content and phrasing of each proposed session. Based on this process, the program content and delivery method were adapted to the target population’s characteristics, drawing on an online support program for significant others of people with depression and an intervention for young people whose parents have a mental illness [30,31]. The vFPE program uses FPE as the core framework and includes supplementary cognitive behavioral therapy (CBT)–informed problem-solving components. The core FPE elements incorporate psychoeducation about MDD and communication skills to support interaction with the family member with MDD, which are consistent with those in previous FPE programs for MDD [12]. However, the current program differs from previous FPE programs in that it also incorporates CBT-informed problem-solving techniques and practical guidance on navigating social and community resources tailored to each participant’s situation. Psychoeducation is expected to enhance caregivers’ understanding of MDD and reduce uncertainty and self-blame. Communication practice is intended to promote more adaptive interactions with the family member with MDD. In addition, drawing on a prior problem-solving intervention for family caregivers of people with bipolar disorder, the vFPE program includes CBT-informed problem-solving components to help caregivers cope with caregiving-related stress [32]. The problem-solving components are expected to enhance coping and self-efficacy. Resource navigation is intended to facilitate access to appropriate support. Through these mechanisms, the intervention is expected to contribute to improvements in caregivers’ anxiety and depressive symptoms, mental well-being, psychological distress, caregiver burden, self-efficacy, and health-related quality of life.

The intervention comprises 4 weekly 40-minute sessions. This structure was chosen to accommodate the lifestyles of young adult caregivers, who often have multiple roles, while minimizing participant burden and ensuring sufficient time for psychoeducation and individualized support. This format is also consistent with previous telehealth-based FPE for family caregivers of people with mental disorders, in which multisession formats, including 40-minute web-based or videoconference-based sessions, were used [33,34]. Sessions will be delivered according to a prespecified intervention manual using educational materials.

The program consists of 4 structured sessions covering psychoeducation about MDD and caregiving experiences, communication with the family member with MDD, stress management using problem-solving and relaxation techniques, and help-seeking and support resources. Table 1 presents the objectives, core content, session activities, homework or between-session tasks, educational materials, and expected provider behaviors for each session.

**Table 1.** Session structure and core components of the videoconference-based family psychoeducation (vFPE) program^a^.

Session	Objective	Core content	Session activities	Homework or between-session task	Educational materials	Expected provider behaviors
Session 1: understanding MDD^b^ and sharing caregiving experiences	To improve understanding of MDD and help participants reflect on their caregiving experiences	Symptoms, course, recurrence, and treatment of MDD; impact of MDD on family members; caregiver burden; and self-care	Provide psychoeducation on MDD, discuss common difficulties and emotional reactions among family caregivers, and invite participants to share their caregiving experiences	Reflect on situations in which interaction with the family member feels difficult or burdensome	Slides on MDD and caregiving; caregiver reflection worksheet	Provide accurate information in plain language, validate the participant’s experiences, and avoid blaming the caregiver or the family member with MDD
Session 2: communication with the family member with MDD	To support adaptive communication with the family member with MDD	Communication strategies, responses to depressive symptoms, and strategies to reduce critical or self-blaming interactions	Review difficult communication situations, discuss alternative responses, and practice supportive communication using examples from the participant’s daily life	Try one communication strategy in daily life and reflect on the experience	Slides on communication strategies, video materials on adaptive communication, and communication practice worksheet	Tailor examples to the participant’s relationship and family context, encourage realistic and nonjudgmental responses, and provide individualized feedback
Session 3: stress management and problem-solving	To enhance coping with caregivers’ own stress	Stress cycle, problem-solving steps, coping strategies, brief relaxation	Identify caregiving-related stressors, apply problem-solving steps to one current difficulty, and practice brief relaxation	Select one stressor and use the problem-solving worksheet and practice relaxation as feasible	Slides on stress management, problem-solving worksheet, and video materials on relaxation	Help the participant break down problems into manageable steps, support feasible coping strategies, and encourage self-care and realistic goal-setting
Session 4: help-seeking and support resources	To promote help-seeking and use of support resources	Information on social support, medical and welfare services, counseling, family associations, and community resources; help-seeking barriers	Review available support resources, discuss barriers to using support, simulate help-seeking, and develop an individualized resource-use plan	Continue using the selected support option or resource-use plan after the program	Resource list and help-seeking planning worksheet	Provide information relevant to the participant’s situation, support autonomous decision-making, and avoid pressuring the participant to use specific services

a Session content may be tailored to each participant’s caregiving situation while maintaining the core components of the intervention.

b MDD: major depressive disorder.

#### Intervention Delivery

The intervention will be delivered via Zoom, a videoconferencing platform. To protect privacy and security, each session will have a passcode and a waiting room enabled, and the provider will verify the participant before admission. Participants will be asked to join each session from a private location where conversations cannot be overheard, and the intervention provider will conduct sessions in a private room at the study institution. If a participant misses a session, it will be rescheduled in consultation with the participant, with a maximum rescheduling window of 2 weeks for each session. All 4 sessions must be completed within 8 weeks of the first session. Participants who do not complete all 4 sessions within the allowable intervention period, including rescheduled sessions, will be recorded as noncompleters.

The intervention manual and session materials may be made available from the corresponding author upon reasonable request, where appropriate.

#### Intervention Fidelity

The vFPE sessions will be conducted in a one-to-one format by a nurse trained in CBT and FPE. The intervention provider has received prior training based on the intervention manual. To protect participant privacy and confidentiality, sessions will not be audio- or video-recorded.

To ensure intervention fidelity, the fidelity checklist was developed based on the intervention manual and previous recommendations for intervention reporting and fidelity assessment [35,36]. The checklist will assess whether the core components of each session were delivered as planned. Consistent with fidelity rating tools used in psychosocial interventions, each item will be rated as 0=not delivered, 1=partially delivered, or 2=delivered as planned [37]. The intervention provider will complete the checklist after each session to document adherence to the intervention manual and any deviations. In addition, the intervention provider will receive regular supervision from an expert in psychiatric care throughout the intervention period to maintain the quality of sessions. The supervisor will review selected session notes and fidelity checklists during supervision. Deviations from the manual will be documented with reasons and reviewed during supervision.

#### Waitlist Control Group

A waitlist control design was adopted due to the lack of standardized psychoeducation programs for young adult caregivers and the ethical need to ensure that all participants had an equal opportunity to receive the intervention.

During the study period, the waitlist control group will continue their usual daily routines and undergo assessments concurrently with the intervention group. Upon completion of the 1-month follow-up assessment, participants in the waitlist control group will be offered the same vFPE as that provided to the intervention group.

#### Concomitant Support

Participants may continue to receive their usual medical care and support throughout the study, with no restrictions on seeking advice from their family member’s physician or other supporters. They will also not be prevented from receiving clinically necessary routine care and consultation.

However, to minimize potential contamination of exploratory clinical outcome comparisons, participants will be asked to refrain from initiating a new structured psychotherapy during the study period. This restriction does not apply to brief daily advice or general consultation support.

### Outcomes

#### Baseline and Clinical Characteristics

Baseline and clinical characteristics will include participants’ age, sex, educational attainment, employment status, marital status, living arrangement, relationship to the family member, time spent providing care, and use of social resources. Additionally, data on the family member with MDD will include age, sex, educational attainment, employment status, marital status, duration of illness, current frequency of outpatient visits, and hospitalization history.

#### Primary Outcomes: Feasibility and Acceptability

##### Recruitment Feasibility

Recruitment feasibility will be primarily assessed by whether the target sample size of 24 participants can be enrolled and randomized within the planned recruitment period. Based on the target sample size and recruitment period, the anticipated recruitment pace is approximately one participant per month. Recruitment progress will be reviewed during the recruitment period, and recruitment strategies will be modified if recruitment is slower than expected. As a supplementary recruitment indicator, the screening-to-randomization rate, defined as the proportion of participants who are randomized among those who receive a study explanation and complete eligibility screening, will also be calculated, with 80% or higher considered acceptable.

Consistent with previous studies [38,39], to capture the recruitment process more broadly, we will record and descriptively summarize the number of individuals reached or approached where available, those who contact the research team, receive a study explanation, provide informed consent, complete eligibility screening, are judged eligible, are randomized, as well as those who decline participation. We will record the consent rate, randomization acceptance, session attendance, the number of missed and rescheduled sessions, videoconferencing problems, questionnaire completion at each assessment time point, and reasons for withdrawal or nonparticipation. These data will be summarized descriptively to assess the feasibility of recruitment, randomization, intervention delivery, and data collection.

##### Intervention Completion Rate

Intervention completion rate will be defined as the proportion of participants allocated to the intervention group who complete all 4 vFPE sessions. The number of sessions attended by each participant will also be summarized descriptively. Intervention completion and session attendance have been used as feasibility indicators in previous brief psychoeducational pilot studies [40,41]. Based on these prior studies and the brief 4-session structure of the vFPE program, an intervention completion rate of 70% or higher will be considered acceptable.

##### Dropout Rate

Dropout rate will be calculated at the postintervention and 1-month follow-up and will be defined as the proportion of randomized participants who discontinue participation in the study or who are lost to follow-up before each assessment time point. The number of dropouts and reasons for dropout will be summarized descriptively. Considering that dropout and retention have been considered important feasibility indicators in previous studies of family caregivers, a dropout rate of 30% or lower at the 1-month follow-up will be considered acceptable [16,42].

##### Safety

Safety will be monitored by recording the occurrence, nature, severity, and outcome of adverse events and serious adverse events throughout the study period. The causal relationship between each adverse event and the intervention will also be assessed.

##### Acceptability

Acceptability will be assessed using the Client Satisfaction Questionnaire-8 Japanese version (CSQ-8-J) among participants in the intervention group at the postintervention assessment after the completion of the 4-session program. The CSQ-8-J is an 8-item self-administered questionnaire rated on a 4-point Likert scale that measures satisfaction with treatment. Total scores range from 8 to 32, with higher scores indicating greater treatment satisfaction [43,44].

##### Progression to a Future Definitive RCT

Progression to a future definitive RCT will be based on an integrated assessment of the prespecified quantitative criteria—recruitment of 24 participants within the planned period, a screening-to-randomization rate of at least 80%, an intervention completion rate of at least 70%, and 1-month follow-up retention of at least 70%—together with randomization acceptance, intervention fidelity, acceptability, videoconferencing problems, and safety. These criteria will be used as guidance rather than as strict rules [45].

Progression without major modifications will be considered if the prespecified feasibility criteria are met and no serious safety concerns are identified. If one or more criteria are not met but the identified problems are considered remediable through procedural modifications, progression with modifications will be considered. Progression will not be recommended if serious safety concerns or major feasibility problems that cannot reasonably be addressed are identified.

### Exploratory Outcomes

All exploratory outcomes will be assessed at 3 time points—baseline, postintervention, and 1-month follow-up—via self-administered questionnaires.

#### Main Exploratory Outcome

The main exploratory outcome is the Hospital Anxiety and Depression Scale (HADS) total score (HADS-T). Anxiety and depressive symptoms were selected as the main exploratory outcome because young adult caregivers may experience emotional distress related to caregiving roles and uncertainty about the illness. The HADS is a 14-item self-administered questionnaire with 2 subscales: anxiety (HADS-A) and depression (HADS-D) [46]. Although the HADS was originally developed for hospital outpatient settings, it has also been examined in nonclinical and family caregiver samples [47,48] and used in previous studies of family caregivers of people with mental illness [49-51]. The Japanese version has demonstrated good reliability and validity [52]. The HADS will be administered at baseline, postintervention, and 1-month follow-up. Scores on the HADS-A and HADS-D will be evaluated as supplementary outcomes, with scores of 8 or higher indicating elevated symptom levels [53]. In this study, the HADS-T will assess anxiety and depressive symptom severity, whereas caregiver burden will be assessed as a separate exploratory outcome.

#### Other Exploratory Outcomes

The Warwick-Edinburgh Mental Well-Being Scale is a 14-item self-administered measure of mental well-being. It focuses on positive affect and psychological functioning and assesses respondents’ status over the preceding 2 weeks. The reliability and validity of the Japanese version have been established [54,55].

The Kessler Psychological Distress Scale is a 6-item self-administered measure of general psychological distress [56]. The Japanese version has also been shown to be reliable and valid, and it is widely used to assess nonspecific psychological distress in diverse populations [57].

The Japanese version of the 8-item Zarit Caregiver Burden Interview is a self-administered measure used to assess caregiver burden in a brief format. The original version was developed by Zarit and colleagues, and the reliability and validity of the Japanese short version have also been demonstrated [58,59]. In this study, it will be used to assess changes in subjective burden associated with family caregiving.

The General Self-Efficacy Scale is a 16-item self-administered measure of general self-efficacy, with higher scores indicating greater general self-efficacy. It will be used to assess individuals’ confidence in coping with challenges in daily life and in initiating actions on their own [60].

The EQ-5D-5L is a self-administered measure used to comprehensively assess health-related quality of life across 5 dimensions: mobility, self-care, usual activities, pain/discomfort, and anxiety/depression. The EQ-5D-5L has demonstrated acceptable psychometric properties across diverse populations, and a Japanese value set has been established [61,62]. In this study, it will be used to assess changes in overall health-related quality of life following the intervention.

### Sample Size

As this study is a pilot trial conducted prior to a future definitive RCT, the sample size was not determined via a formal power calculation. Instead, the target sample size was set at 12 participants per group (N=24).

This aligns with a commonly cited rule of thumb suggesting that approximately 12 participants per group may be appropriate for pilot studies [63]. This sample size may also provide preliminary data to inform the planning of a future definitive RCT [64].

If 9 out of 12 intervention-group participants complete the intervention, the completion rate will be 75.0% (95% CI 46.8%‐91.1%). If 17 of 24 participants complete the 1-month follow-up, the retention rate will be 70.8% (95% CI 50.8%‐85.1%). Recruiting 24 participants over 24 months would correspond to 1.0 participant per month (95% CI 0.64‐1.49). Because the CIs for completion and retention cross the prespecified 70% thresholds, these thresholds will guide rather than determine progression decisions. Despite the limited precision, these estimates can identify major feasibility problems and inform a future definitive RCT.

### Randomization

Upon eligibility confirmation and completion of the baseline assessment, participants will be randomly assigned in a 1:1 ratio to either the intervention or the waitlist control group. Randomization will be performed via UMIN INDICE Cloud (University Hospital Medical Information Network Center, The University of Tokyo Hospital), using the minimization method. Allocation factors include sex and age (<25 y vs ≥25 y). The 25-year age cutoff was selected based on prior literature regarding emerging adulthood and young adult caregivers. Age was selected as an allocation factor to balance the distribution of participants in earlier and later stages of young adulthood across groups [3,65].

The principal investigator will provide the study explanation and obtain written informed consent. A researcher independent of intervention delivery, outcome assessment, and statistical analysis will then register participants whose eligibility has been confirmed by the principal investigator and who have completed the baseline assessment. Following registration, the same researcher will perform centralized allocation via the UMIN INDICE Cloud. As the system generates group assignments only after participant registration, the group assignment will remain unknown to the intervention provider until allocation is completed. The allocation result will then be communicated only to the personnel necessary for intervention scheduling and delivery. Owing to the nature of the intervention, neither participants nor the intervention provider will be blinded.

### Data Collection and Management

Outcome data will be collected at 3 time points: baseline, postintervention, and 1-month follow-up, via online self-administered questionnaires. The schedule of enrollment, intervention, and assessments, including the timing of each outcome measure, is presented in Table 2. To maintain separation between intervention delivery and data management, data collection and management will be handled by a data manager independent of the intervention team. All collected data will be deidentified before storage and management.

**Table 2.** Trial schedule.

Time point	Enrollment	Baseline assessment	Intervention	Postintervention assessment	1-month follow-up assessment
	Time 0	Time 1		Time 2	Time 3
Enrollment					
Informed consent	✓				
Eligibility screening	✓				
Allocation		✓			
Interventions					
vFPE^a^			✓		
Waitlist			✓		
Assessments					
Demographic information		✓			
Family information		✓			
Outcome measures					
HADS^b^		✓		✓	✓
WEMWBS^c^		✓		✓	✓
K6^d^		✓		✓	✓
J-ZBI_8^e^		✓		✓	✓
GSES^f^		✓		✓	✓
EQ-5D-5L		✓		✓	✓
Feasibility outcomes					
Recruitment feasibility	✓				
Intervention completion rate			✓		
Dropout rate				✓	✓
Adverse events			✓	✓	✓
Intervention acceptability					
CSQ-8-J^g^^,^^h^				✓	

a vFPE: videoconference-based family psychoeducation.

b HADS: Hospital Anxiety and Depression Scale.

c WEMWBS: Warwick-Edinburgh Mental Well-Being Scale.

d K6: Kessler Psychological Distress Scale.

e J-ZBI_8: Japanese version of the 8-item Zarit Caregiver Burden Interview.

f GSES: General Self-Efficacy Scale.

g CSQ-8-J: Client Satisfaction Questionnaire-8 Japanese version.

h CSQ-8-J will be administered to the intervention group only.

To maximize retention and completion of 1-month follow-up assessments, participants will receive reminder emails or messages before each relevant intervention session and assessment time point. Furthermore, when necessary, flexible scheduling will be offered to accommodate participants’ needs and facilitate participation in the intervention sessions.

### Statistical Analysis

The reporting of study results will follow the CONSORT (Consolidated Standards of Reporting Trials) extension for randomized pilot and feasibility trials [66]. Exploratory outcomes will be analyzed according to randomized group allocation using available outcome data at each follow-up time point. Participant characteristics will be summarized using means and SDs for continuous variables and frequencies and percentages for categorical variables.

The primary outcomes, feasibility and acceptability, will be summarized descriptively. Recruitment pace will be reported as participants per month, while screening-to-randomization, intervention completion, and dropout rates will be reported as proportions with 95% CIs. Safety will be summarized descriptively based on the occurrence and characteristics of adverse events and serious adverse events. Acceptability will be summarized descriptively using CSQ-8-J scores among intervention-group participants with available postintervention CSQ-8-J data. Scores will be categorized as poor (8-13), fair (14-19), good (20-25), and excellent (26-32) [67].

Exploratory outcomes will be analyzed to obtain preliminary estimates of between-group differences and variability, rather than for formal hypothesis testing. Descriptive within-group changes, between-group differences in change scores, and 95% CIs will be presented. For HADS-T, the main exploratory comparison will be the between-group difference at postintervention. The between-group difference at the 1-month follow-up will be examined as a supplementary exploratory comparison.

Between-group differences at each follow-up time point will be estimated using a baseline-adjusted ANCOVA model. The dependent variable will be the HADS-T score at each follow-up time point. The model will include group as the independent variable, with baseline HADS-T, sex, and age category included as covariates. Sex and age category will be included as covariates because they are used in the minimization procedure [68].

Other exploratory outcomes will be analyzed using the same model structure, with the baseline score for each respective outcome, sex, and age category included as covariates. We will report adjusted mean differences, 95% CIs, and standardized effect sizes corrected for small-sample bias using Hedges *g*. Each follow-up analysis will include participants with available outcome data at that time point. Missing data will be summarized by group and time point, and no imputation of missing outcome data will be performed. No adjustment for multiplicity will be applied because all analyses of exploratory outcomes are intended to be preliminary. Statistical analyses will be performed using R software (R Foundation for Statistical Computing).

As this is a pilot trial not intended to provide a definitive test of effectiveness, all inferential results will be interpreted as exploratory. The main purpose of the clinical outcome analyses is to obtain preliminary estimates of outcome variability to inform the design of a future definitive RCT. Due to the small sample size, no subgroup or sensitivity analyses will be performed in this pilot trial.

### Data Monitoring

As this is a minimal-risk pilot trial, an independent data monitoring committee will not be established, and external auditing will not be conducted. Instead, study progress and safety will be monitored by the principal investigator and coinvestigators through the regular review of recruitment, retention, intervention delivery, adverse events, and data collection. No formal interim analyses are planned because of the small sample size and pilot nature of the trial. There are no prespecified statistical stopping guidelines. However, if serious safety concerns arise, the principal investigator, in consultation with the coinvestigators and the ethics committee as needed, will determine whether the trial should be modified or terminated.

### Adverse Event Assessment

Adverse events will be monitored during intervention sessions and at the postintervention and 1-month follow-up assessments. Assessment items will include increased psychological distress, worsening depressive or anxiety symptoms, heightened study-related burden, and other mental or physical problems.

If marked deterioration is observed, the research team will determine the appropriateness of continued participation and, if necessary, discontinue the intervention or withdraw the participant from the study. In such instances, participants will also be encouraged to seek appropriate medical care or support services. All adverse events will be recorded, and any serious adverse events will be promptly reported to the ethics committee.

### Ethical Considerations

This study will be conducted with the approval of the Research Ethics Committee, Faculty of Nursing, Toho University (approval number RE2025016). The trial was registered in the Japan Registry of Clinical Trials (jRCT; trial ID jRCT1032250574) on December 17, 2025, prior to the commencement of participant enrollment.

Individuals interested in participating will receive a comprehensive written and verbal study explanation via videoconferencing. Written informed consent will be obtained by the principal investigator before the initiation of any study procedures. As all participants are adults aged 18 years or older, parental consent will not be required. Participants will be informed that their participation is voluntary and that they may withdraw from the study at any time without penalty. Signed consent forms will be stored securely, separately from the study data, in either a locked cabinet or a password-protected electronic environment accessible only to authorized research personnel.

Participants who complete all assessments will receive a QUO card worth JP¥ 5000 (approximately US $33) as compensation for their time and efforts. Any significant protocol modifications will be reviewed by the research team and submitted to the Research Ethics Committee, Faculty of Nursing, Toho University for approval prior to implementation. Trial registration details in the Japan Registry of Clinical Trials will also be updated accordingly. If necessary, participants will be informed of the changes, and additional consent will be obtained.

## Results

This paper describes the study protocol. Ethical approval was obtained from the Research Ethics Committee, Faculty of Nursing, Toho University, on December 18, 2025. Recruitment commenced on January 10, 2026, and the first participant was enrolled on February 20, 2026. As of May 2026, 8 participants had been enrolled. Intervention delivery and data collection are expected to continue until March 2028. Study findings will be disseminated through presentations at academic conferences and publication in peer-reviewed journals.

## Discussion

### Expected Findings

This pilot trial will evaluate the feasibility of conducting a waitlist-controlled RCT of vFPE for young adult caregivers of a family member with MDD. Although young adult caregivers often have substantial support needs, they remain an underserved population because they must balance caregiving with other developmental and social responsibilities, including employment, education, partnership, and parenting.

This study has 2 potential strengths. First, it will contribute to the limited evidence on supportive interventions for young adult caregivers of a family member with MDD, a population that has received relatively little attention despite their distinct developmental and psychosocial challenges. Second, it will examine the feasibility of conducting a waitlist-controlled RCT of vFPE and the acceptability of this brief videoconference-based intervention specifically designed to mitigate the time-related and geographic barriers to accessing support prevalent in this population.

These findings are expected to inform the design of a future definitive RCT. Recruitment indicators and sources will be used to refine strategies for future trials. Data on dropout, intervention completion, and acceptability will inform potential modifications to the intervention content and intervention delivery procedures. Estimates of outcome variability and findings from exploratory outcomes will inform the design of a future trial.

### Limitations

This study also has several limitations. First, as this is a small pilot trial, it is not designed to provide a definitive evaluation of intervention effectiveness; therefore, findings related to clinical outcomes should be interpreted as exploratory, and generalizability is limited. Second, all exploratory clinical outcome measures are based on participant self-report, and participant blinding is not feasible; therefore, the possibility of expectancy bias cannot be excluded. Third, data regarding the diagnosis and clinical history of the family member with MDD will be based solely on participant-reported information and will not be verified using medical records. Although caregiver-reported diagnostic information has been used in previous intervention studies involving caregivers of individuals with depression [14,69], relying on participant-reported information to identify psychiatric diagnoses may reduce diagnostic accuracy [70]. Diagnostic uncertainty or misclassification may remain if the diagnosis has changed over time or if comorbid psychiatric conditions are present. Fourth, excluding participants currently receiving outpatient treatment for a mental disorder may limit the generalizability of the findings to young adult caregivers receiving mental health treatment. This limitation is important because previous research has found that more than half of caregivers of patients with depressive disorder had a psychiatric diagnosis [49]. Therefore, caregivers with substantial mental health support needs may be underrepresented in this study. Finally, the requirement of internet access and the ability to complete online procedures may introduce selection bias, favoring individuals who have greater digital access or literacy.

### Conclusions

This pilot trial will clarify the feasibility of conducting an RCT of vFPE for young adult caregivers of a family member with MDD. The findings are expected to provide preliminary evidence to inform the design of a future definitive RCT and advance the development of accessible support for this underserved population.

## Supplementary material

10.2196/105182 Checklist 1 SPIRIT 2025 checklist.

## Abbreviations

CBT: cognitive behavioral therapy
CONSORT: Consolidated Standards of Reporting Trials
CSQ-8-J: Client Satisfaction Questionnaire-8 Japanese version
FPE: family psychoeducation
HADS: Hospital Anxiety and Depression Scale
HADS-A: Hospital Anxiety and Depression Scale–Anxiety subscale
HADS-D: Hospital Anxiety and Depression Scale–Depression subscale
HADS-T: Hospital Anxiety and Depression Scale total score
MDD: major depressive disorder
RCT: randomized controlled trial
SPIRIT: Standard Protocol Items: Recommendations for Interventional Trials
vFPE: videoconference-based family psychoeducation
